# The Neuro-Ecology of *Drosophila* Pupation Behavior

**DOI:** 10.1371/journal.pone.0102159

**Published:** 2014-07-17

**Authors:** Francisco Del Pino, Claudia Jara, Luis Pino, Raúl Godoy-Herrera

**Affiliations:** Programa de Genetica Humana, Instituto de Ciencias Biomedicas, Facultad de Medicina, Universidad de Chile, Santiago, Chile; Center for Genomic Regulation, Spain

## Abstract

Many species of *Drosophila* form conspecific pupa aggregations across the breeding sites. These aggregations could result from species-specific larval odor recognition. To test this hypothesis we used larval odors of *D. melanogaster* and *D. pavani*, two species that coexist in the nature. When stimulated by those odors, wild type and *vestigial* (*vg*) third-instar larvae of *D. melanogaster* pupated on conspecific larval odors, but individuals deficient in the expression of the odor co-receptor *Orco* randomly pupated across the substrate, indicating that in this species, olfaction plays a role in pupation site selection. Larvae are unable to learn but can smell, the *Syn^97CS^* and *rut* strains of *D. melanogaster*, did not respond to conspecific odors or *D. pavani* larval cues, and they randomly pupated across the substrate, suggesting that larval odor-based learning could influence the pupation site selection. Thus, *Orco*, *Syn^97CS^* and *rut* loci participated in the pupation site selection. When stimulated by conspecific and *D. melanogaster* larval cues, *D. pavani* larvae also pupated on conspecific odors. The larvae of *D. gaucha*, a sibling species of *D. pavani*, did not respond to *D. melanogaster* larval cues, pupating randomly across the substrate. In nature, *D. gaucha* is isolated from *D. melanogaster*. Interspecific hybrids, which result from crossing *pavani* female with *gaucha* males clumped their pupae similarly to *D. pavani*, but the behavior of *gaucha* female x *pavani* male hybrids was similar to *D. gaucha* parent. The two sibling species show substantial evolutionary divergence in organization and functioning of larval nervous system. *D. melanogaster* and *D. pavani* larvae extracted information about odor identities and the spatial location of congener and alien larvae to select pupation sites. We hypothesize that larval recognition contributes to the cohabitation of species with similar ecologies, thus aiding the organization and persistence of *Drosophila* species guilds in the wild.

## Introduction

No animal lives in isolation. Animals interact with one another in many different ways using their sensory systems. Olfaction and gustation help to detect, localize and recognize congeners and heterospecifics, and provide information regarding food availability and the chemical features of environments where animals live [1]–[11]. Holometabolous insect larvae possess sophisticated olfactory and gustatory receptors and brain structures that process a variety of olfactory and gustatory inputs [1]–[9], [12], [13], suggesting the importance of these sensory systems for the ecology and evolution of these individuals. Larva behavior is relevant to understand certain aspects of holometabolous insect population biology, including spatial distributional patterns, interactions between congeners and alien individuals, and feeding preferences. However, in *Drosophila* there are considerable gaps in our understanding of larva behavior because the ecology of natural breeding sites in which neurological organization functions is poorly known. Additional studies are needed because several investigations have suggested that pupation site selection depends on discrimination of chemicals emitted by conspecifics and alien larvae [8], [14], [15]. Pupation behavior is important for fitness because it implies habitat use and plays a role in the coexistence of species with similar ecologies. Experiments addressing these problems provide insight into how the nervous system generates behaviors related to recognition between conspecific and alien larvae, and how the behaviors are genetically specified and linked to neural circuits. These two aspects are key to understand the evolution of behavior in the genus *Drosophila*.

To address the above problems we observed the behavior of *Drosophila* larvae in nature. We also identified species cohabiting within decaying fruits (grape, apple, peach and prickly pear) and cactus cladodes (*Opuntia ficus-indica*). We noted that many *Drosophila* species formed conspecific pupa aggregations across the breeding sites away from the pupae of other species (see Figures S1 and S2). These observations suggest that larvae can recognize congeners and alien larvae. Namely, larvae appear to associate spatial position of congeners with favorable locations to pupate while eluding pupae and/or larvae of other species.

*Drosophila* breeding sites are ephemeral and variable and their ecological conditions can change quickly [16]. Microbial action on sugars in commercial fruits as apple, peach, and prickly pear, and on plant tissues produces variable concentrations of odors composed of alcohols, esters and some fatty acids [10], [17], [18]. In these circumstances, species-specific larval odors could act as indicators of spatial position of conspecific and alien larvae. Namely, these chemical cues would provide information on identity and emission source, thus orienting larval movements within the breeding sites. Identifying congeners would be more efficient if the process was coupled with social odor learning. For individuals who reside in changing environments, learning has a profound effect on their fitness [9]. Reviews of the factors that contribute to the emergency of learning emphasize the role played by changing habitats [19], [20]. Namely, learning is favored by natural selection when environmental changes occur in a short lapse of time, and those changes are recurring [21]. Learners may increase their fitness acquiring behaviors similarly to those expressed by other members of the species. These behaviors will be adaptive if they are appropriate for the current environmental state [22]. The relationship between pupation behavior in ephemeral breeding resources and social odor learning in *Drosophila* has received little attention. Previously, we observed that *Drosophila simulans* and *Drosophila buzzatii* larvae that bred with congeners formed pupa aggregations, but larvae that were individually reared in isolation from the conspecifics pupated randomly across the substrata [8], [14]. These results suggested larval social odor learning. Here we describe pupation behavior of *D. melanogaster* mutant larvae that cannot smell and cannot learn via conspecific and *D. pavani* larval odors. In Chile, these two species use decaying fruits of *Opuntia ficus-indica* as breeding sites.

However, there are also closely related *Drosophila* species that differ in their physiological ability to use different foods, and this may have consequences for the larva behavior [23]. Larvae of those species that exploit a range of breeding sites have more opportunities to be reared with other *Drosophila* species than larvae of ecologically restricted species [16]. The presence/absence of other species might be an evolutionary pressure to *Drosophila* larval nervous system. *D. pavani* and *Drosophila gaucha* are allopatric sibling species in the *mesophragmatica* group that reside in Chile and Argentina, respectively [24]–[26]. In Chile, *D. pavani* and *D. melanogaster* emerge from decaying prickly pear fruits. In Argentina, *D. gaucha* breeds on decaying prickly pear cladodes (tissue). *D. melanogaster* larvae die when they are transferred to decaying cladodes (unpublished data). *D. gaucha* dies when it is bred on prickly pear fruit. Thus, *D. gaucha* and *D. melanogaster* larvae are ecologically isolated. We investigated pupation behavior of *D. pavani* and *D. gaucha* stimulated by conspecific and *D. melanogaster* larval cues.

## Materials and Methods

### Subjects

We tested wild type larvae of natural Chilean populations (Til-Til and Trana strains) and laboratory stocks (Oregon R-c and Canton – Special strains) of *D. melanogaster*. We also examined larvae of the *vestigial* (*vg*) strain. The Oregon R-c and *vg* strains differ in some larval behaviors. For example, Oregon R-c larvae dig deeper into the substratum than *vg* larvae [27]. We also tested three neurological mutants derived from the Canton-Special (CS) strain of *D. melanogaster*. Larval perception of odorants in the *Orco* mutant strain has been blocked because the dendritic localization of the receptors has been eliminated. Thus, the *Orco* mutation disrupts behavioral and electrophysiological responses to many odorants [29]. The *Syn^97CS^* mutation affects presynaptic vesicle release in the entire larval brain, and olfactory associative learning is reduced in approximately 50% with respect to CS larvae; however, responsiveness to stimuli and motor performance in untrained animals are normal [28]. The *rut* locus participates in olfactory conditioning learning in *D. melanogaster* and it is expressed in neurons located in larval and adult mushroom bodies; *rut* does not affect larval locomotion and responsiveness to stimuli [30].

In 1952, Professor Danko Brncic brought the Oregon R-c and *vg* strains from Columbia University (USA) to the University of Chile. Dr Bertram Gerber, University of Würzburg in Germany sent the Canton-Special (CS), *Orco*, *Syn^97CS^* and *rut* strains to our laboratory. The wild type Trana strain of *D. melanogaster* was established with 22 adults that emerged from 20 decaying grains of grape (*Vitis vinifera*, País variety); Trana is located 380 km southwest of Santiago. The wild type Til-Til strain of *D. melanogaster* was formed with 18 adults that emerged from three decaying prickly pear fruits collected in Til-Til, which is 50 km northwest of Santiago. The Til-Til and Trana larvae in the laboratory experiments were the fourth generation.

To investigate larval olfactory recognition in other *Drosophila* species, we examined the endemic South American sibling species *D. pavani* and *D. gaucha* (Subgenus *Drosophila*, *mesophragmatica* group) and the F_1_ reciprocal hybrids [24]–[26]. The *mesophragmatica* group comprises many South American species and forms a phyletic unit [24]. *D. pavani* is predominantly Andean in distribution, whereas *D. gaucha* is distributed in Argentina, Uruguay and Southern Brazil. Both species share characteristics with domesticated species in that the adults can be collected from orchards, garden, and other plants associated with human activities [26]. Under laboratories conditions, the two species can produce viable but sterile hybrids [31]. *D. pavani* and *D. gaucha* have similar development durations for molting, wandering and pupating [25]. *D. pavani* and *D. gaucha* require approximately one and half year to assimilate to laboratory conditions. For this reason we tested the La Florida and Buenos Aires strains (see below).

In 1978, Professor Danko Brncic founded the La Florida strain of *D. pavani* with approximately 18 individuals collected from banana traps in La Florida, Santiago (Chile). Same prickly pear plants remain in this neighborhood today. In 1989, Dr Esteban Hasson sent the Buenos Aires strain of *D. gaucha* to us. This strain was created with 10 adults collected from banana traps in Buenos Aires (Argentina). The traps were established near prickly pear plants. The sex ratio was variable for the all strains.

The strains were all maintained by mass culture at 24±1°C, 70% humidity (*D. melanogaster*) and 18±1°C, 80% humidity (*D. pavani* and *D. gaucha*). *D. pavani* and *D. gaucha* grow better at this temperature and humidity than at 24°C. All stocks were maintained under constant light, because facilities to change the light/dark period were not available in the laboratory.

### Crosses

Fifteen-day-old *D. pavani* (La Florida strain) and *D. gaucha* (Buenos Aires strain) males and females were reciprocally crossed. At this age, individuals are sexually competent [24]. Homogametic mating within strains also served as controls for the interspecific crosses. Crosses between the La Florida (*D. pavani*) and Buenos Aires (*D. gaucha*) strains provided abundant hybrid larvae of the two sexes [31].

### Larva collection

Groups of 40–50 inseminated females of *D. melanogaster*, *D. pavani*, *D. gaucha*, and *D. pavani* females and *D. gaucha* females crossed with males of the other species were allowed to oviposit for 2–3 h on plastic spoons containing the culture medium. Thirty eggs of the species, strains and hybrids were randomly collected with a dissecting needle. Each batch of eggs was incubated on fresh spoons for 96–100 h at 24°C (*D. melanogaster* strains) and 168–172 h at 18°C (*D. pavani*, *D. gaucha* and the hybrids). *D. melanogaster* larvae emerged after 2–4 h. *D. pavani*, *D. gaucha* and hybrid larvae emerged 48 h after the eggs were laid. The culture medium was supplemented daily with 40% fresh baker’s yeast paste. One hour before an experiment, third-instar larvae were collected from the glass wall of rearing vials, washed twice with distilled water, and identified by the presence of protruded anterior spiracles [32]. All larvae were raised in half-pint bottles at 24°C (*D. melanogaster*) and 18°C (*D. pavani* and *D. gaucha*) on Burdick’s medium [33].

### Larval odor recognition and pupation site selection

We employed three treatments to test the response of *D. melanogaster* larvae to conspecific and *D. pavani* larval odors. In the first treatment, one 2.0×2.0 cm piece of Whatman cellulose filter paper was moistened for 1 h in Burdick’s medium used for 4–5 days by *D. melanogaster* (or *D. pavani*) larvae, whereas another identically sized filter paper was moistened in sterile Burdick’s medium for a similar period of time. Before transferring the two filter paper types to Petri dishes, each piece of paper was carefully examined under stereomicroscope to verify that no food was adhered to the surface. In the second treatment, one piece of paper was impregnated for 1 h with food worked by larvae of the strain (Canton-Special, Til-Til, Trana, *vestigial*, *Orco*, *Syn^97CS^* and *rut* strains of *D. melanogaster*). The other filter paper was moistened for a similar time with food processed by the Oregon R-c larvae. The test for larvae of this strain was an Oregon R-c filter paper, and a Canton – Special filter paper. In the third treatment, the larvae were placed together with a filter paper that had been moistened for 1 h in food occupied by larvae of *D. melanogaster* strains, and another moistened for a similar time with the food used by *D. pavani* larvae. These treatments were also applied to *D. pavani*, *D. gaucha* and the interspecific reciprocal hybrids.

For each treatment, 10-cm Petri dishes were filled with 10 ml of 3% agar gel. Each filter paper type was deposited onto agar in Petri dishes on opposite sites at 6 cm one from the other. Batches of 20 third-instar larvae of each species and strain were introduced into corresponding Petri dish and gently deposited onto the middle of the agar 3 cm from each piece of paper. Once the larvae actively moved, we transferred the Petri dishes to the culture room maintained at 24±1°C (*D. melanogaster*), and 18±1°C (*D. pavani*, *D. gaucha* and the hybrids). To decrease possibilities that temperature differences and illumination conditions would interfere with pupation site preferences, we always deposited the Petri dishes in the identical location in the culture room. The number of pupae on/under each paper was recorded two (*D. melanogaster*), four–five days later (*D. pavani*, *D. gaucha* and the hybrids). Some pupae were observed on the agar surface near the paper. Those individuals detected within 10 mm of the border were counted as belonging to that paper. Replicate measurements (10 measurements, 200 larvae) were performed for each strain by dividing the larvae into groups of 5 replicates and conducting the measurements in parallel. Thus, we were able to estimate magnitude of environmental odor variation on larval response to chemical cues. We did not know whether the substances present in the paper could diffuse through the agar, which might have introduced an additional source of experimental error to our measurements because of possible larval gustatory responses. To address this variable, we tested *Orco* mutant larvae. There were no odor-evoked responses for the specialized olfactory sensory neurons of *Orco* larvae. The *Orco* mutation does not affect gustatory neurons [29].

We also monitored the pupa distributions on the agar in the virgin food/*D. pavani* food and virgin food/Oregon R-c food treatments. In the first treatment, wild type and *vg* pupae of *D. melanogaster* were clumped across the agar, whereas *Orco, Syn^97CS^* and *rut* pupae were randomly distributed. In the virgin food/Oregon R-c food treatment, wild type, *vg*, *Orco, Syn^97CS^* and *rut* pupae were scattered across the agar. We calculated the Clark and Evans aggregation index [34] to compare the pupa distributions in the two treatments. We estimated the average distance to the nearest neighboring (rA) pupa using a 0.5-cm Cartesian grid and then compared this average value with the expected value (rE) for the identical number of individuals randomly distributed on an area of equal size (rE = ½√ρ), where ρ is the pupa density. The ratio R = rA/rE, reflects the spatial distribution of the individuals (aggregated, random, over-dispersed) with values ranging between R = 0.0 (maximum aggregation) and R = 2.15 (uniform). When individuals are randomly distributed, R = 1.0 [34]. We followed a similar procedure for the pupa distributions of *D. pavani* (La Florida strain), *D. gaucha* (Buenos Aires strain) and the reciprocal interspecific hybrids in the virgin food/*D. pavani* (*D. gaucha*, hybrid) food, and virgin food/Oregon R-c food treatments.

All behavioral tests were performed between 10∶00 and 14∶00 hours under controlled temperature, 22.00±1.0°C, light, 6.2±0.2×10^−5^ lx, and humidity, 70±3%.

### Statistical analysis

We tested for homogeneity between replicates within a treatment and strain. We applied a *G*-test of Independence to compare the pupa percentages: N = 10 replicates per strain, N = 8 strains of *D. melanogaster*, N = 3 treatments. The test was also applied to the pupa percentages obtained for replicated experiments: N = 10 replicates within a treatment, N = 2 treatments, with *D. pavani*, *D. gaucha*, and hybrid larvae, N = 4 genotypic groups. None of the individual chi-square tests detected a significant deviation from the hypothesis of homogeneity between the replicates within a strain or treatment (the Chi-square values were all lower than the critical value χ^2^ = 23.59, *df* = 9; *P*>0.05; Table S1). Thus, we pooled the replicate data within treatments, strain, and species.

We also applied a binomial test to the data. The null hypothesis stated that there was no difference between the probability of selecting one filter paper type and the probability of selecting another paper within a treatment or strain (p = q = ½). The binomial test was selected because the data were in two discrete categories and the experimental design utilized one sample (see above homogeneity between replicates within a treatment and strain and Table S1). The rejection region of the null hypothesis was equal or less than α = 0.01.

An analysis of variance was applied to compare pupa aggregation indexes (R) of *D. melanogaster* in the presence and absence of *D. pavani* larval volatiles. The analysis was also applied to pupa aggregations of *D. pavani*, *D. gaucha* and the hybrids stimulated by *D. melanogaster* larval volatiles.

## Results

### Pupation site selection: *D. melanogaster*

**Figure 1:**
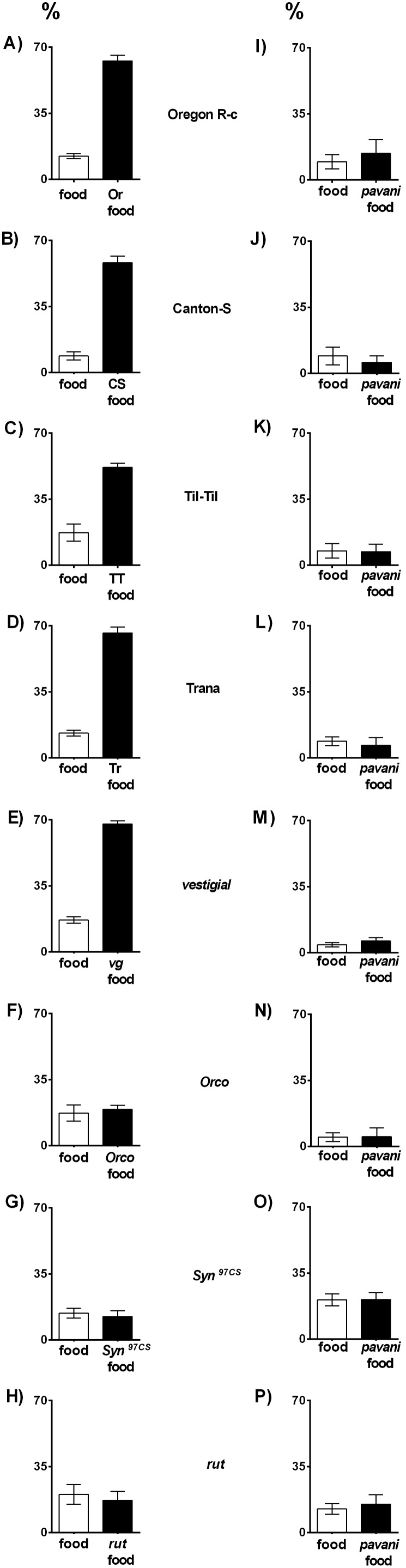
Pupation behavior of third-instar larvae *D. melanogaster* stimulated by the odors emanating from filter paper moistened with virgin food, food processed by conspecific larvae, and food worked by *D. pavani* larvae. Preferences are shown as percentage of pupae ± SE on the papers, N = 10 replicates, 200 larvae per strain. White column (**A**–**P**), filter paper moistened with virgin food. Black column, filter paper moistened with food worked by larvae of the strain (**A**–**H**) and *D. pavani* larvae (**I**–**P**). The strains tested were: Oregon R-c (Or) and Canton-Special (CS), wild type, laboratory stocks, and Til-Til (TT) and Trana (Tr), wild type, natural populations. Mutant strains were also tested: *vestigial* (*vg*); *Orco*, dendritic localization of odorant receptors is abolished; *Syn^97CS^* learning mutant, deletion for phosphoproteins in presynaptic terminals; *rut* learning mutant, encodes a calmodulin dependent adenylate cyclase that converts ATP to cyclic AMP. Statistical significance is in Table S2.

More than 60% of wild type and *vg* pupae of *D. melanogaster* were on the conspecific paper, thus indicating that they were stimulated by odors emanating from the two types of paper one moistened with sterile food and the other with food processed by larvae of the own strain (Fig. 1 **A–E**). Differences in pupa percentages on the two paper types are statistically important (Table S2). By contrast, confronted with paper moistened with virgin food and food worked by *D. pavani* larvae more than 60% of the wild type and *vg D. melanogaster* pupae were located on the agar, and approximately 17% were located on the two paper types (Fig. 1 **I–M**, and Table S2). Clearly, the wild type and *vg D.* m*elanogaster* larvae recognized and responded to conspecific and *D. pavani* chemical cues. In the two treatments, more than 60% of the *Orco* pupae were scattered across agar (Fig. 1 **F–N**, and Table S2), thus suggesting that the *Orco* larvae did not detect the odors emitted from the two paper types. In a similar way, stimulated by conspecific and *D. pavani* larval odors, *Syn^97CS^* and *rut* larvae also were randomly distributed across agar (Fig. 1 **N–P**, and Table S2). Thus, the *Orco*, *Syn^97CS^* and *rut* loci are necessary for pupation site selection in *D. melanogaster.*

**Figure 2:**
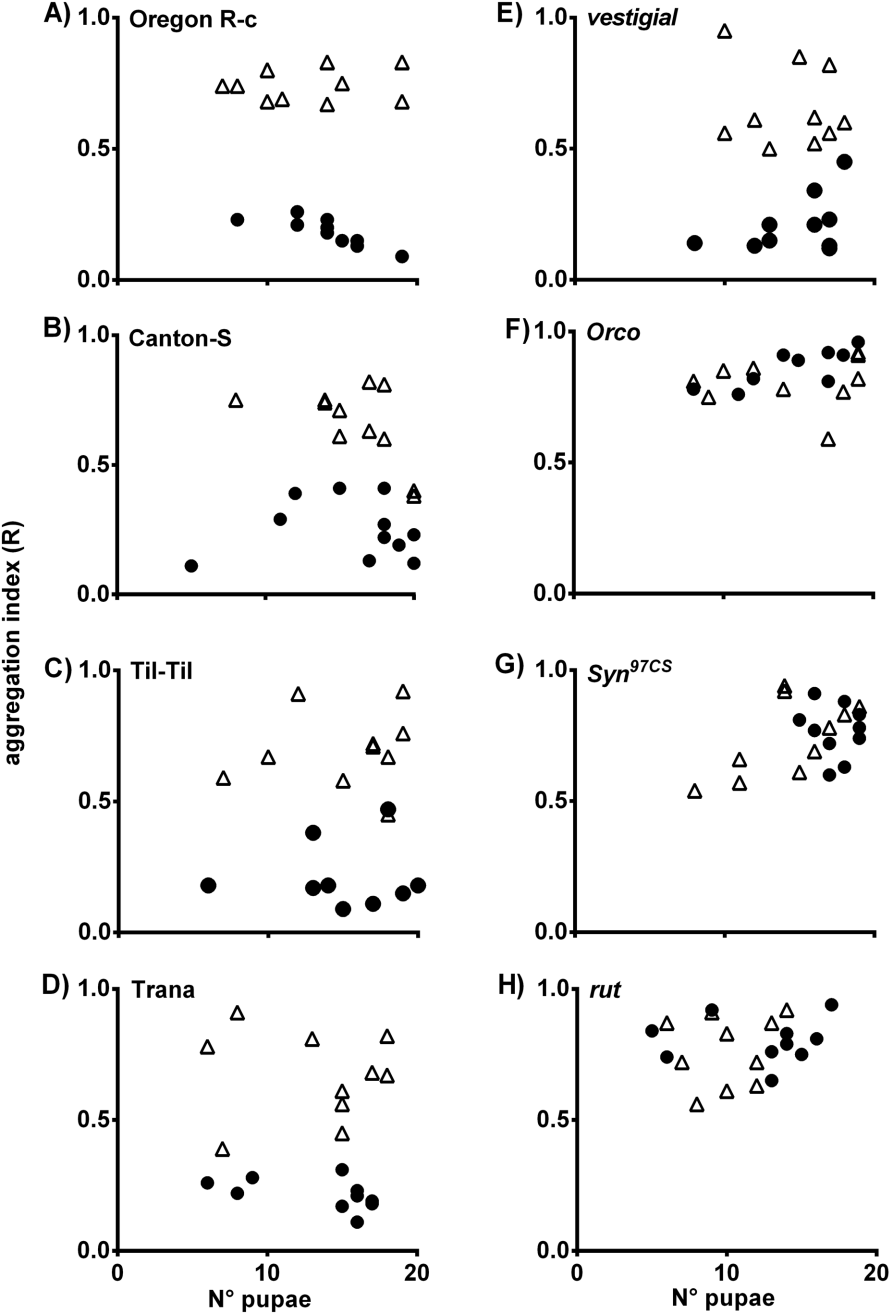
Pupa aggregations indexes (R) and number of pupae on agar of the Oregon R-c (A), Canton-Special (B), TIl-Til (C), Trana (D), *vestigial* (*vg*) (E), *Orco* (F), *Syn^97CS^* (G), and *rut* (H) strains of *D. melanogaster*. White triangle indicate aggregation in the presence of conspecific larval odors; black circle, aggregation in the presence of *D. pavani* larval odors. R = 1.0 indicates that individuals are randomly distributed over substrates. R = 0.0, maximum aggregation. For statistical significance, see Table S3.

The pupa aggregation (R) indexes were calculated for the wild type and mutant strains of *D. melanogaster*. In the virgin food/*D. pavani* food treatment (Fig. 2 **A–H**), the aggregation R-values for the wild type and *vg* strains of *D. melanogaster* were approximately R = 0.0, which indicated grouped pupae (Fig. 2 **A–E**, black circles). In the presence of chemicals emanating from the two types of papers moistened with virgin food and Oregon R-c food, the calculated R-values were approximately R = 1.0, which indicated that the pupae were randomly distributed across the substrate (Fig. 2 **A–E**, white triangles). These differences in the pupa aggregation indexes were statistically important (ANOVA, Table S3). In the presence of conspecific and alien larval odors, the pupa aggregation (R) indexes of the *Orco*, *Syn^97CS^* and *rut* strains were approximately R = 1.0, which confirmed that the larvae pupated randomly across the agar (Fig. 2 **F–H**, and Table S3). The results again indicated that these three loci are necessary for *D. melanogaster* pupation behavior. For all strains tested, the aggregation indexes were independent of the number of pupae on the agar (Fig. 2 **A–H**).

**Figure 3:**
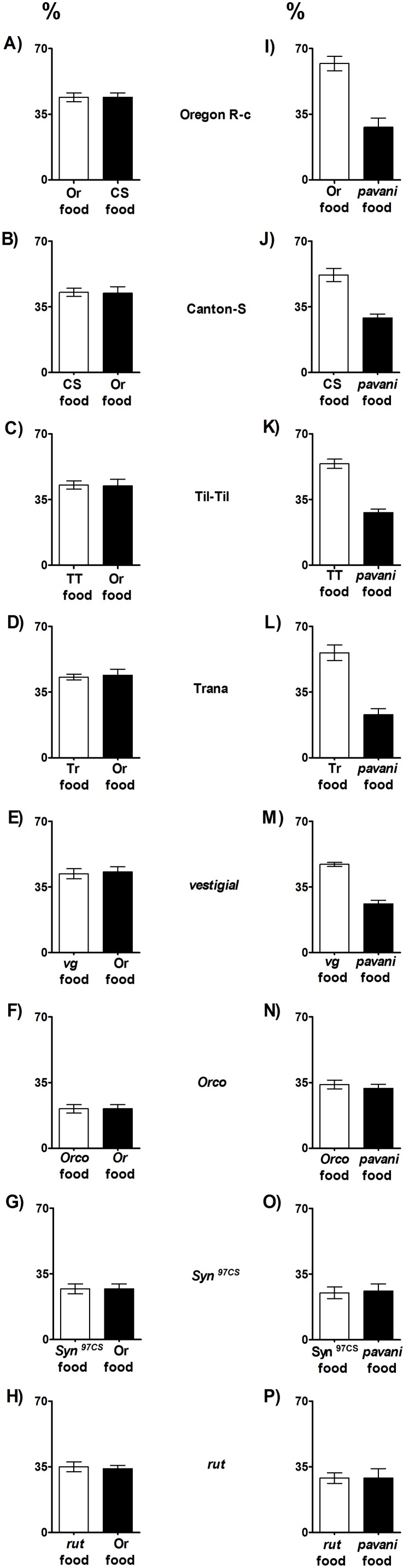
Pupation site selection in the presence of conspecific and *D. pavani* larval cues in *D. melanogaster* larvae. **A–H**; white column, filter paper moistened with food used by larvae of the strain; black column, paper moistened with Oregon R-c food. **I–P**, white column, filter paper moistened with food of the strain; black column, paper moistened with food processed by *D. pavani* larvae. For details, see Figure 1; statistical analysis in Table S3.

**Fig. 3** **. A–E** shows that *D. melanogaster* larvae that were stimulated by conspecific odors used the two types of paper to pupate. However, in the presence of conspecific and *D. pavani* larval cues, the wild type and *vg D. melanogaster* pupae were preferentially observed on the conspecific paper (Fig. 3 **I–M**; Table S2). In the two treatments, similar percentages of *Orco*, *Syn^97CS^* and *rut* pupae were found on the two paper types (Fig. 3 **F–H** and **N–P**; Table S2). These results and those in Figs. 1 and 2 again indicate that in *D. melanogaster* olfaction is required for pupation site selection and that genes expressed in the neurons of the peripheral olfactory circuits [29], synapses [11], and mushroom bodies [2] participate in *D. melanogaster* pupation behavior.

Pupae were also detected outside the two paper types on the Petri dish agar. In the treatment with two types of conspecific food (Fig. 3 **A–H**), a few pupae were present on agar. The calculated (R) aggregation indexes (N = 10 replicates) were (i) Oregon R-c, 0.88±0.02; (ii) Canton-Special, 0.83±0.09; (iii) Til-Til, 0.70±0.04; (iv) Trana, 0.91±0.05; (v) *vg*, 0.79±0.06; (vi) *Orco*, 0.81±0.08; (vii) *Syn^97CS^*, 0.95±0.10; and (viii) *rut*, 0.97±0.05. In the conspecific food/*D. pavani* food treatment (Fig. 3 **I–P**), 79±2.31% of the wild type and *vg D. melanogaster* pupae were adhered one another, as observed in nature (Figs. S1 and S2). In these two identical treatments, the *Orco*, *Syn^97CS^* and *rut* pupae were scattered across the substrate. These results are consistent with those in Figs. 1–3.

### Pupation site selection: the sibling species *D. pavani* and *D.gaucha*

**Figure 4:**
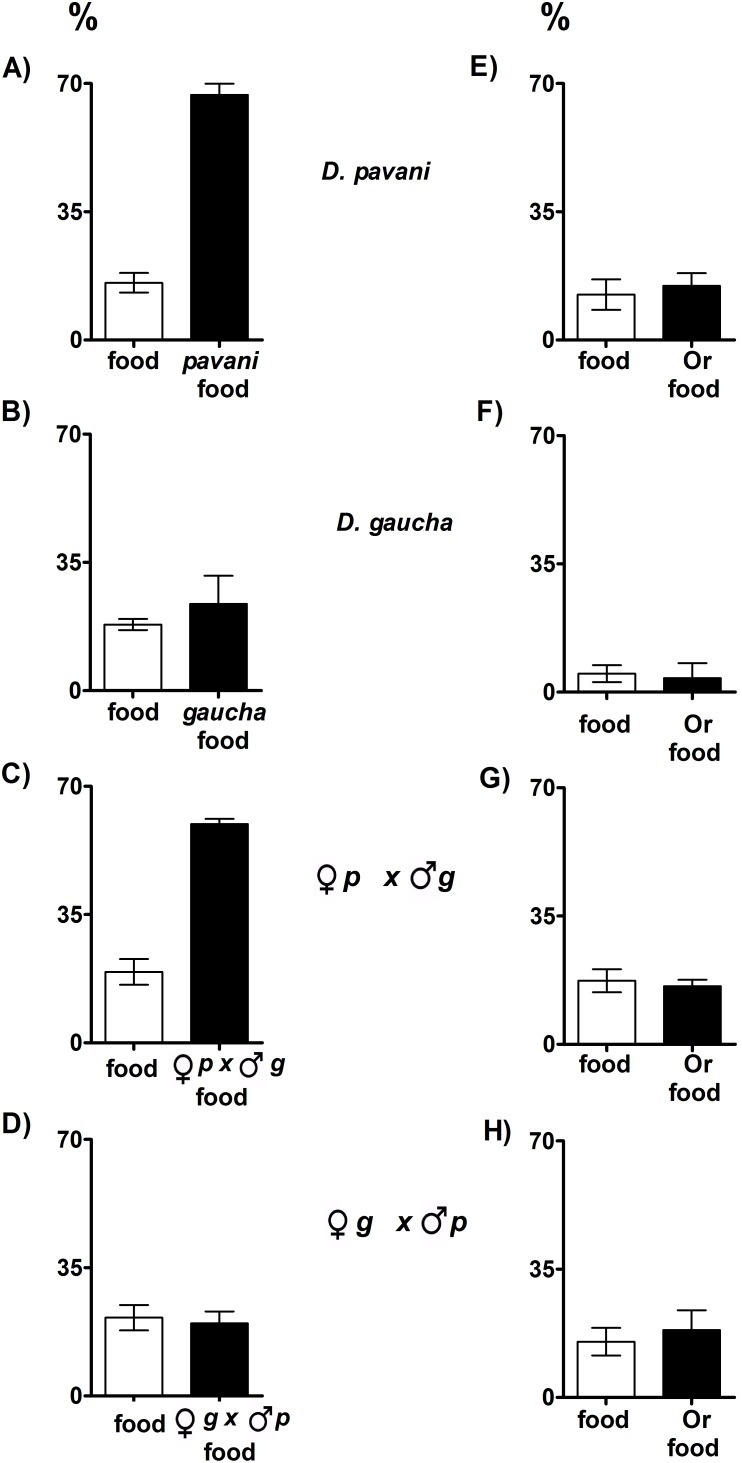
*D. pavani*, *D. gaucha* and the hybrids pupation site preferences. The larvae were stimulated by signals emitted from a paper moistened with virgin food and a paper moistened with food worked by conspecific larvae (**A–D**), and from a paper moistened with virgin food and a paper moistened with the Oregon R-c strain (**E–H**). For details, see Figure 1; statistical analysis in Table S2.

In the virgin food/conspecific food treatment, approximately 68% of *D. pavani* and the *pavani* female x *gaucha* male hybrid larvae pupated on the conspecific paper (Fig. 4 **A**, **C**, Table S2). In this identical situation, *D. gaucha* and the *gaucha* female x *pavani* male hybrid larvae used both paper types to pupate (Fig. 4 **B**, **D**, Table S2). Notably, more than 70% of the pupae of the four groups of genotypes were located on the agar, and less than 14% were located on the paper types moistened with virgin food and food processed by *D. melanogaster* larvae (Fig. 4 **E–H**, Table S2).

**Figure 5:**
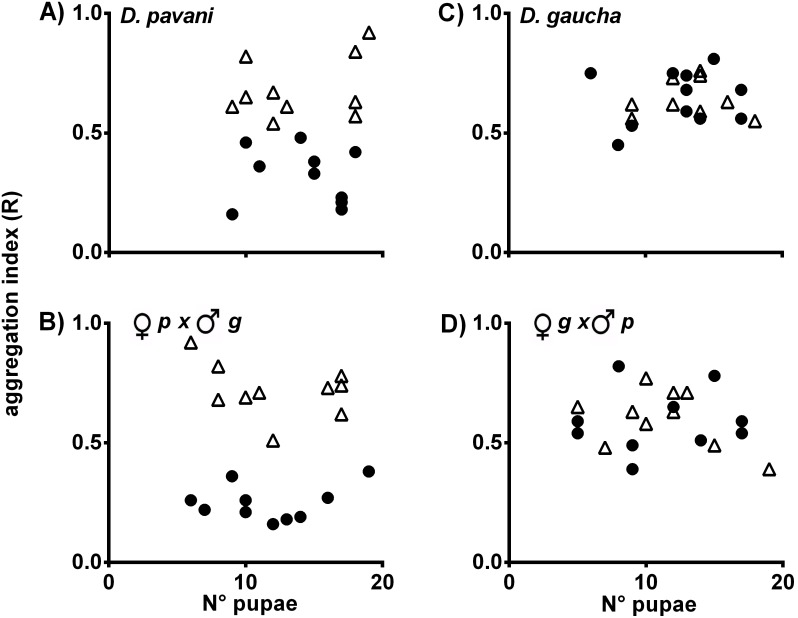
Pupa aggregation indexes (R) and number of pupae on agar of *D. pavani* (A), *D. gaucha* (C), and the interspecific hybrids (B, D). White triangle aggregation in the absence of *D.melanogaster* larval cues; black circle aggregation in the presence of *D. melanogaster* larval signals. For details, see Figures 1 and 2; statistical analysis in Table S3.

**Figure 5** **. A–D** shows that *D. pavani* and the *pavani* female x *gaucha* male hybrid larvae that were stimulated by *D. melanogaster* larval volatiles pupated near congeners (Fig. 5 **A**, **B**, black circles). Aggregation decreased in the presence of conspecific larval chemicals (Fig. 5 **A**, **B**, white triangles). These differences between the two treatments were statistically important (Table S3). The results confirmed that olfaction is needed for pupation site selection in *D. pavani* and *pavani* female x *gaucha* male hybrid larvae. By contrast, in the presence of conspecific and *D. melanogaster* larval cues, *D. gaucha* and *gaucha* female x *pavani* male hybrids randomly pupated across the agar (Fig. 5 **C**, **D**, white triangles and black circles), which suggested that these larvae did not use olfaction to select their pupation sites.

**Figure 6:**
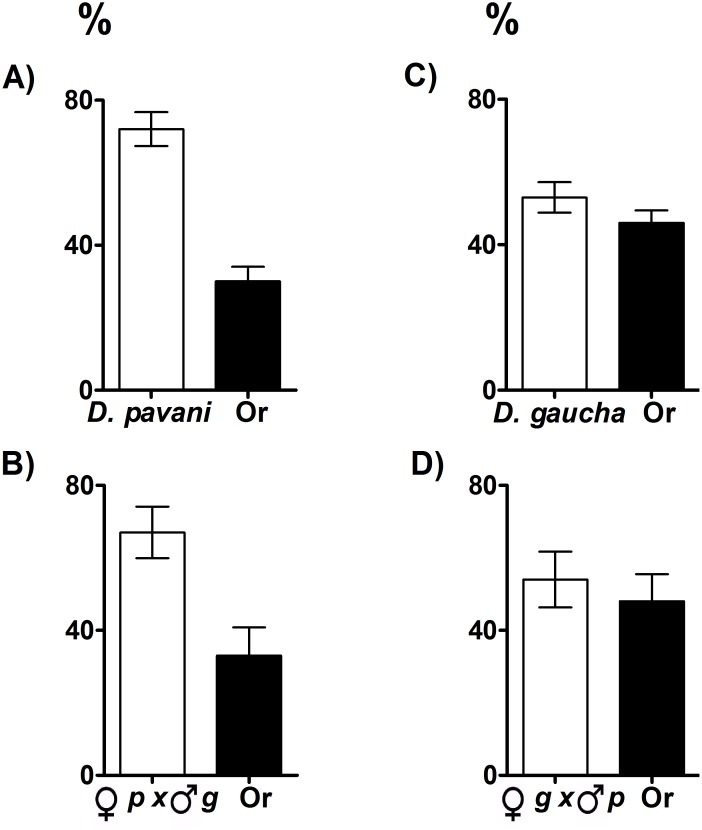
*D. pavani* (A), *D. gaucha* (C) and the interspecific hybrids (B, D) pupation site preferences. Preferences are expressed as percentages ± SE of pupae on a paper moistened with food worked by conspecific larvae, white column, and on a paper moistened with food processed by *D. melanogaster* larvae (the Oregon R-c (Or) strain) paper, black column. For details, see Figure 1; statistical analysis in Table S2.

Stimulated at the same time by conspecific and *D. melanogaster* larval cues, *D. pavani* and *pavani* female x *gaucha* male hybrid larvae pupated more on conspecific target paper than *D. melanogaster* paper (Fig. 6 **A**, **B**, Table S2). However, a similar percentage of *D. gaucha* and *gaucha* female x *pavani* male hybrid pupae were detected on the two paper types (Fig. 6 **C**, **D**, Table S2). Clearly, in the two sibling species divergent evolutionary changes in the organization and function of the larval nervous system involved in pupation behavior have occurred.

## Discussion

*D. melanogaster* and *D. pavani* third-instar larvae emit, perceive and react to conspecific and alien larval volatiles, influencing pupation behavior. Larvae of the two species pupate near congeners and away from pupae of other species. Confronted with *D. pavani* larval odors, *D. melanogaster* increased pupa aggregation by decreasing the distance between nearest neighboring conspecific pupae. However, stimulated by their own odors, the distance between pupae increased, thus decreasing the aggregation. *D. pavani* larvae exhibit behavior comparable to that of *D. melanogaster*. These plastic features of pupation behavior in the two species are consistent with the fact that they coexist in the same orchards [15], [25], [26], [35]. *D. buzzatii* and *D. simulans* also showed flexible pupation behaviors and increased pupa aggregation in the presence of alien larval odors [14], [15]. *D. simulans* and *D. melanogaster* belong to the subgenus *Sophophora*, *melanogaster* subgroup; *D. buzzatii* belongs to the subgenus *Drosophila*, *repleta* group, *buzzatii* cluster; and *D. pavani*, belongs to the subgenus *Drosophila*, *mesophragmatica* group [36]. We conclude that olfaction is required for pupation site selection in many *Drosophila* species.

However, our results also indicated that *D. gaucha*, the sibling species of *D. pavani*, did not use larval odor input to select their pupation sites. These behavioral differences are most likely because of the ecological differences between the two sibling species. In Argentina, *D. gaucha* is reared on cladodes of *Opuntia ficus-indica* in the absence of *D. melanogaster* and *D. pavani* ([24]–[26], and personal observations). In Chile, *D. pavani, D. melanogaster* and *D. simulans* emerge from decaying prickly pear fruits (unpublished data). *D. gaucha* die when bred on prickly pear fruits. On the other hand, *D. melanogaster* does not survive on prickly pear cladodes (unpublished data). Thus, in nature, larvae of the two species do not share identical breeding sites. These ecological differences could explain why *gaucha* larvae do not respond to *melanogaster* larval volatiles. The deprivation of odor experience may lead to a loss of sensitivity and acuity for distinguishing odorants [37]. We expect that *D. melanogaster* and *D. pavani* larvae do not respond to the larval volatiles of *D. gaucha*. It is also possible that *D. gaucha* does not emit larval odors.

### *D. melanogaster*

*D. melanogaster* has a shorter larval period than *D. pavani* - 4 to 6 days versus 8 to 12 days, at 24°C - [32]. This difference may mean that *D. melanogaster* larvae searching for pupation sites do not encounter *D. pavani* larvae and/or pupae. However, this cannot account for why the third-instar larvae of the two species modified their pupation behavior when stimulated by the odors of the other species. Perhaps conspecific and alien larvae identification occurs when first and second instar larvae are feeding within the breeding sites. Therefore, it is important to investigate whether first and second instar larvae of *Drosophila* associate larval odors with the presence of conspecific and alien larvae. Further studies are planned to investigate this possibility.

The chemicals present in the paper used could diffuse through the agar. Thus, larval gustatory responses could be also involved in our measurements. Nevertheless, in the presence of conspecific and *D. pavani* larval odors, the *Orco* larvae pupated randomly across the agar, and distance between nearest neighboring *Orco* pupae did not change. These mutant larvae were without the *Orco* odor co-receptor but had gustatory ones [29]. The *Orco* mutation interferes with larval odor recognition, thus affecting pupation behavior. These results confirm that olfaction, not gustation, is essential for pupation site selection in *D. melanogaster*.

On the other hand, stimulated by conspecific odors and *D. pavani* odor cues, *Syn^97CS^* and *rut* larvae also pupate randomly across the Petri dish agar. Thus, these two mutations cause notable changes in the pupation behavior of *D. melanogaster*. These two loci affect olfactory associative learning in *D. melanogaster* [28], [30]. We suggest that larval odor-based learning could be involved in the pupation behavior of *D. melanogaster*. *D. buzzatii* and *D. simulans* larvae reared in isolation from conspecifics pupate randomly across the substrate, but form pupa aggregations when reared with congeners [14]. Notably, larvae of *Drosophila hydei*, subgenus *Drosophila*, *repleta* group, *hydei* subgroup and *Drosophila busckii*, subgenus *Dorsilopha* also form pupa aggregations away from *D. melanogaster* and *D. pavani* pupae [15]. These results suggest that social larval odor-based learning could be an integral component of pupation site selection and it could be widely extended throughout the genus *Drosophila*.

Larvae of many *Drosophila* species develop within unpredictable, heterogeneous and rapidly changing breeding environments [8], [16]–[18], [35]. Fermentation increases the acidity of fruits, and microbial action also produces alcohols, esters and other odoriferous, toxic substances that cumulate within the breeding sites surrounding the larvae [14], [17], [18]. On the other hand, decaying fruits are exposed to desiccation reducing their availability as breeding sites. These are all heterogeneous processes because some sections of a fruit may be in an advanced state of dryness and decay, whereas desiccation and fermentation are only beginning in other sections of the fruit. The presence of other species of larvae within the identical decaying fruit unit is another difficult variable to predict. In Chilean orchards three to four *Drosophila* species may emerge from a fruit, whereas only one species may emerge from the identical fruit type located 15–18 cm away (unpublished data). Thus, many biotic and abiotic variables cause important ecological changes to which *Drosophila* larvae must respond. In these changing environments, larval odor-based learning could be important to pupation behavior [21]. The spatial positions on the breeding sites where conspecifics emit odor signals could be associated with favorable locations to pupate. Alien larval odors could be indicative of an unfit area colonized by the larvae of other species. *Orco*, *Syn^97CS^* and *rut* mutations interfere with the association process by causing disturbances and blocking the recognition of odors of congeners and alien larvae. Thus, *Orco*, *Syn^97CS^* and *rut* loci are crucial for *D. melanogaster* pupation behavior because they influence (i) the spatial orientation of larval movements, (ii) the identification of conspecifics and (iii) the ability to form groups away from alien larvae.

### *D. pavani* and *D. gaucha*

Notably, when stimulated by *D. melanogaster* larval cues, *D. pavani* pupae were grouped, but *D. gaucha* pupae were observed scattered across the substrate. Thus, *D. gaucha* larvae show a markedly different behavior to that of *D. pavani* larvae. Namely, *D. gaucha* larvae did not appear to use the olfactory system to select their pupation sites. However, these conjectures could be not completely correct. Fig. 4 **B** shows that approximately 35% of *D. gaucha* pupae were present on the conspecific and sterile food impregnated paper, but in the sterile food/Oregon R-c food treatment, 10% of the pupae were located on the paper (Fig. 4 **F**). These results may be indications that *D. gaucha* larvae detected *D. melanogaster* larval volatiles. However, as in nature, *D. gaucha* was ecologically isolated from *D. melanogaster*, this result could have occurred because of a delay in processing and transmitting information to central structures. In *D. melanogaster*, larval olfactory responses may change according to the post-eclosion odor experience [37]. Further experiments will be required to clarify these results.

### *D.pavani* x *D.gaucha* hybrids

The pupation behavior of the *D. pavani* x *D. gaucha* hybrids confirms that the larvae of the two species differed remarkably in their olfactory sensitivity. The *pavani* female x *gaucha* male hybrid larvae used olfaction to select their pupation sites, but the *gaucha* female x *pavani* male hybrid larvae did not do. However, in the presence of *D. melanogaster* larval cues, the *pavani* female x *gaucha* male hybrid pupae were aggregated, but the *gaucha* female x *pavani* male hybrids pupated randomly across the substrate. These results indicate that in *D. pavani* and *D. gaucha* genes on the X chromosome may have important behavioral effects.

Alternative alleles appear to be localized on the X chromosome of each species. Reciprocal differences in behavior in crosses between the species could also reflex cytoplasmic heredity. Relatively little attention has been paid to olfactory responses in intra-and interspecific hybrid *Drosophila* larvae. These investigations may provide key information about the neurogenetics and evolution of behavior in sibling species of *Drosophila*. Although our experiments demonstrate significant differences in odor responsiveness between *D. pavani* and *D. gaucha*, definitive evidence will require an analysis of chemosensory receptors in larvae of the two species.

Our study suggests that in species of *Drosophila* that coexist in the wild and belong to phylogenetically distant clades, such as *D. melanogaster*, subgenus *Sophophora*, and *D. pavani*, subgenus *Drosophila*, olfaction is used for selection of pupation sites. In *D. gaucha* which is phylogenetically close to *D. pavani*, the larvae do not coexist with *D. melanogaster* or *D. pavani* and do not require olfactory signals to select pupation sites. Thus, our results emphasize that the presence of larvae from two or more *Drosophila* species at breeding sites is significant to understand the role of olfactory system and most likely larval odor-based learning in pupation site selection. Our investigation also emphasizes the significance of genotype, thus providing some information about the genetics of neural circuits underlying to pupation behavior.

In summary, *Drosophila* larva pupation behavior contributes to the partition of space between species with similar ecologies. This behavior has implications for a stable coexistence of many *Drosophila* species in nature. Pupation behavior also helps to organize *Drosophila* communities so that they persist on ephemeral and fragmented breeding sites. The larvae appear to produce some pheromones similar to those observed in adults [16]. In *Drosophila*, imagoes produce cuticular pheromones that chemically correspond to unsatured long-chain hydrocarbons, and the position of double bonds is key for recognize partners. In *D. melanogaster*, males produce abundant monoenes, such as 7- tricosene and 7-pentoacosene; whereas females produce 7, 11-heptacosadiene and 7, 11-nonacosadiene [38]. Some components of salivary gland secretions might function as pheromones in the larvae. Further studies will be necessary to support this hypothesis.

## Supporting Information

Figure S1 **Aggregation of pupae observed on cladodes de prickly pear (** ***Opuntia ficus-indica*** **). Pupae correspond to** ***D. buzzatii*** **(** ***repleta*** **group).**
(JPG)

Figure S2 **Aggregation of pupae observed on grape (** ***Vitis vinifera*** **, País variety). Pupae correspond to** ***D. melanogaster*** **(** ***melanogaster*** **subgroup).**
(JPG)

Table S1 **Chi-square values for differences between replicates within a strain for the indicated species and treatments, degree of freedom = 9.** For the all strains of *D. melanogaster*, the strain x food/Or food interaction (Fig. 3 **A**–**H**) yielded Chi-square values near 1.00.
(DOC)

Table S2 **Probabilities associated with proportions of pupa on the papers in the indicated treatments (the binomial test).** The null hypothesis is no difference between the probability to select one type of paper and the probability to select the other type of paper within a treatment and strain (p = q = ½). Probabilities in the Table indicate whether the null hypothesis must be rejected. The null hypothesis (H_o_) is the pupae are distributed at random over the two papers. When probabilities are smaller than α = 0.01, the decision is to reject H_o_ in favor of H_1_, that is, p>q. For the all strains of *D. melanogaster*, the strain x food/Or food interaction (Fig. 3 **A–H**) yielded probabilities greater than α = 0.01, that is p = q = ½. For other details, see Table S1.
(DOC)

Table S3 **Analysis of variance for pupa aggregation of** ***D. melanogaster*** **expressed as index (R) of aggregation in the presence and in the absence of** ***D. pavani*** **larval volatiles (data in** **Figure 2** **).** Pupa aggregation of *D. pavani*, *D. gaucha* and the hybrids in the presence and in the absence of *D. melanogaster* larval volatiles are also compared (data in Figure 5). F_0.01(1, 9)_ values and their probabilities are shown.
(DOC)
