# Supplementary figures and images for: The Neuro-Ecology of Drosophila Pupation Behavior
Source: PLoS One. 2014 Jul 17;9(7):e102159. doi: 10.1371/journal.pone.0102159 (PMC4102506; doi:10.1371/journal.pone.0102159)

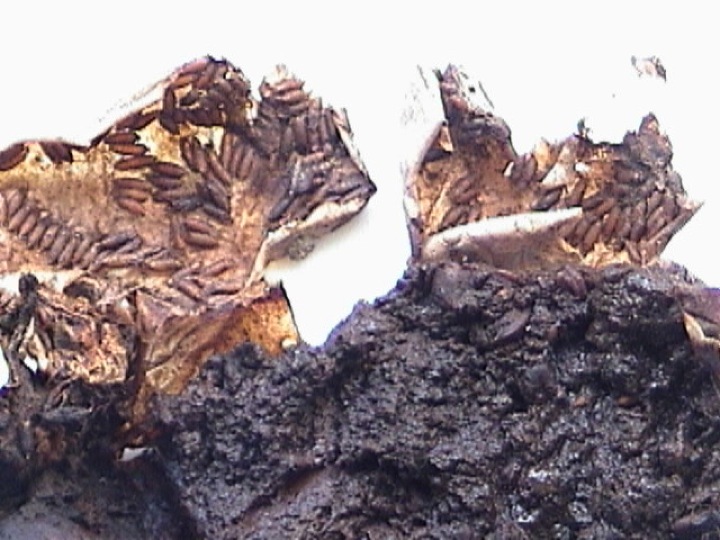

Supplement: Figure S1 — Aggregation of pupae observed on cladodes de prickly pear ( Opuntia ficus-indica ). Pupae correspond to D. buzzatii ( repleta group). (JPG) [file pone.0102159.s001.jpg]

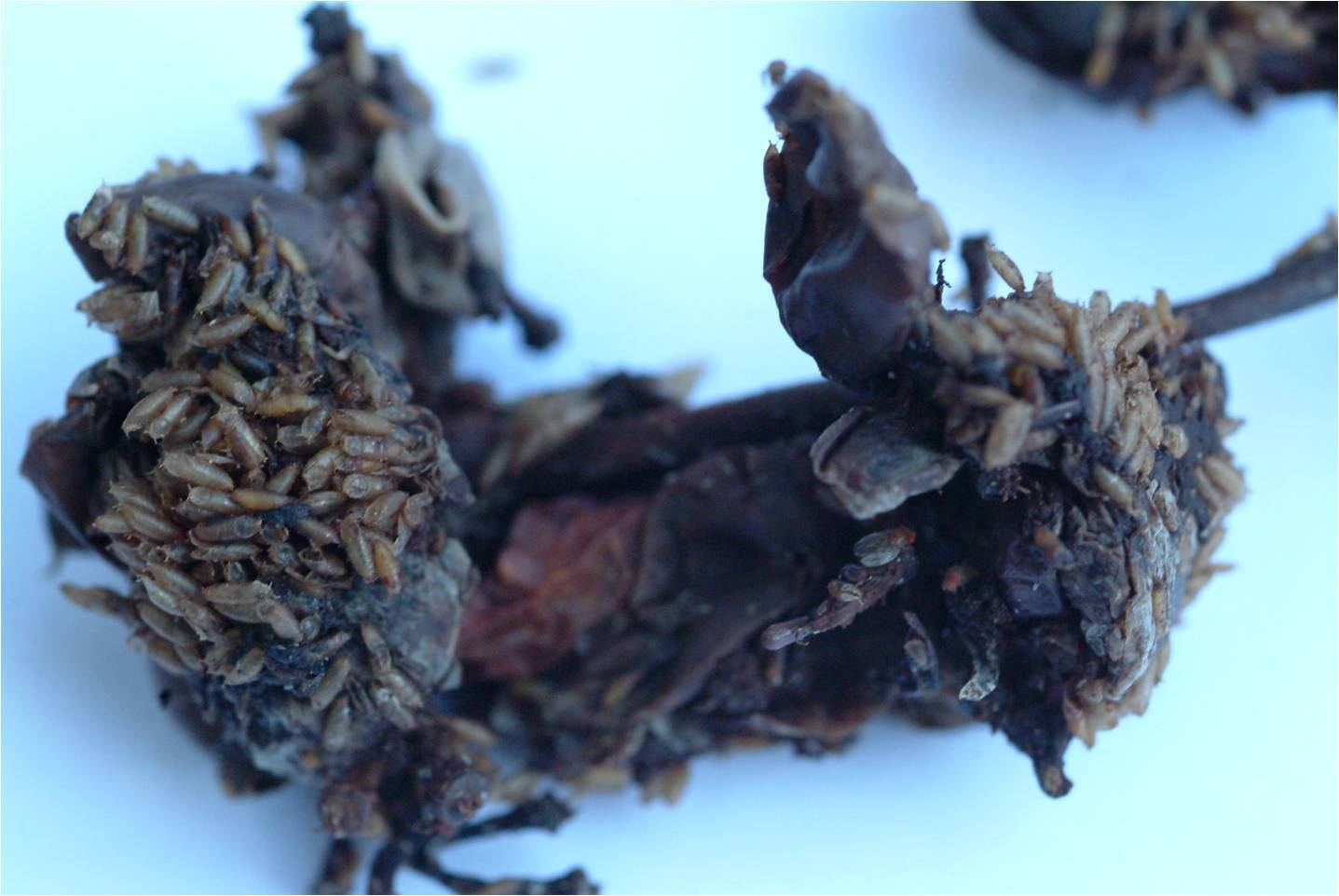

Supplement: Figure S2 — Aggregation of pupae observed on grape ( Vitis vinifera , País variety). Pupae correspond to D. melanogaster ( melanogaster subgroup). (JPG) [file pone.0102159.s002.jpg]
