# Supplementary material for: The Neuro-Ecology of Drosophila Pupation Behavior
Source: PLoS One. 2014 Jul 17;9(7):e102159. doi: 10.1371/journal.pone.0102159 (PMC4102506; doi:10.1371/journal.pone.0102159)
Supplement: Table S1 — Chi-square values for differences between replicates within a strain for the indicated species and treatments, degree of freedom = 9. For the all strains of D. melanogaster, the strain x food/Or food interaction (Fig. 3 A–H) yielded Chi-square values near 1.00. (DOC) [file pone.0102159.s003.doc]

Table S1. Chi-square values for differences between replicates within a strain for the indicated species and treatments, degree of freedom = 9. For the all strains of *D. melanogaster*, the strain x food/Or food interaction (Fig. 3 **A – H**) yielded Chi-square values near 1.00

----------------------------------------------------------------------------------------------------

Species Treatment 1 Treatment 2 Treatment 3

and virgin food/strain food virgin food/*pavani* food strain food/*pavani* food

strain χ2 *P* χ2 *P* χ2 *P*

-------------------------------------------------------------------------------------------------------------------------

*D. melanogaster*

Wild type strains

Oregon R-c 8.43 0.491 5.34 0.804 4.39 0.884

Canton- Special 7.57 0.578 4.09 0.905 7.68 0.567

Til-Til 10.12 0.341 3.21 0.955 3.24 0.954

Trana 9.56 0.387 3.68 0.931 2.67 0.976

Mutant strains

*vestigial* 9.32 0.408 2.67 0.976 2.83 0.971

*Or83b* 3.45 0.944 1.26 0.999 4.58 0.869

*Syn^97CS^* 4.01 0.911 3.04 0.963 2.06 0.990

*rut* 7.31 0.605 4.56 0.871 3.41 0.946

Treatment 1 Treatment 2 Treatment 3

food*/species (or hybrid) food food*/ Or food Or food***/species (or hybrid) food

*D. pavani*

La Florida 11. 02 0.274 8.41 0.493 4.91 0.842

*D. gaucha*

Buenos Aires 13.21 0.153 6. 76 0.662 3.41 0.946

*D. pavani* x *D. gaucha* hybrids**

*pavani x gaucha* 9.67 0.378 4.71 0.859 6.21 0.719

*gaucha x pavani* 8.63 0.472 8.52 0.483 5.37 0.801

*food = virgin food; **for all crosses, the first parent shown is the female; ***Or food = The Oregon R-c food
