# Supplementary material for: The Neuro-Ecology of Drosophila Pupation Behavior
Source: PLoS One. 2014 Jul 17;9(7):e102159. doi: 10.1371/journal.pone.0102159 (PMC4102506; doi:10.1371/journal.pone.0102159)
Supplement: Table S2 — Probabilities associated with proportions of pupa on the papers in the indicated treatments (the binomial test). The null hypothesis is no difference between the probability to select one type of paper and the probability to select the other type of paper within a treatment and strain (p = q = ½). Probabilities in the Table indicate whether the null hypothesis must be rejected. The null hypothesis (Ho) is the pupae are distributed at random over the two papers. When probabilities are smaller than α = 0.01, the decision is to reject Ho in favor of H1, that is, p>q. For the all strains of D. melanogaster, the strain x food/Or food interaction (Fig. 3 A–H) yielded probabilities greater than α = 0.01, that is p = q = ½. For other details, see Table S1. (DOC) [file pone.0102159.s004.doc]

Table S2.- Probabilities associated with proportions of pupa on the papers in the indicated treatments (the binomial test). The null hypothesis is no difference between the probability to select one type of paper and the probability to select the other type of paper within a treatment and strain (p = q = ½). Probabilities in the Table indicate whether the null hypothesis must be rejected. The null hypothesis (H_o_) is the pupae are distributed at random over the two papers. When probabilities are smaller than α = 0.01, the decision is to reject H_o_ in favor of H_1_, that is, p > q. For the all strains of *D. melanogaster*, the strain x food/Or food interaction (Fig. 3 **A – H**) yielded probabilities greater than α = 0.01, that is p = q = ½. For other details, see Table 1.

-------------------------------------------------------------------------------------------------------------------------------------

Species Treatment 1 Treatment 2 Treatment 3

and virgin food/strain food virgin food/*pavani* food strain food/*pavani* food

strain

------------------------------------------------------------------------------------------------------------------------------------*D. melanogaster*

Wild type strains

Oregon R-c 0.001 0.132 0.001

Canton – Special 0.001 0.132 0.001

Til –Til 0.006 0.412 0.001

Trana 0.001 0.412 0.001

Mutant strains

*vestigial* 0.006 0.252 0.001

*Or83b* 0.868 0.252 0.225

*Syn97CS* 0.252 0.500 0.252

*rut*  0.588 0.412 0.252

Treatment 1 Treatment 2 Treatment 3

food/species (or hybrid) food food/ Or food Or food/species (or hybrid) food

*D. pavani*

La Florida 0.001 0.026 0.006

*D. gaucha*

Buenos Aires 0.113 0.041 0.480

*D. pavani* x *D. gaucha* hybrids

*pavani x gaucha*** * 0.001 0.113 0.001

*gaucha x pavani*** * 0.113 0.113 0.670
