# Supplementary material for: The Neuro-Ecology of Drosophila Pupation Behavior
Source: PLoS One. 2014 Jul 17;9(7):e102159. doi: 10.1371/journal.pone.0102159 (PMC4102506; doi:10.1371/journal.pone.0102159)
Supplement: Table S3 — Analysis of variance for pupa aggregation of D. melanogaster expressed as index (R) of aggregation in the presence and in the absence of D. pavani larval volatiles (data in Figure 2 ). Pupa aggregation of D. pavani, D. gaucha and the hybrids in the presence and in the absence of D. melanogaster larval volatiles are also compared (data in Figure 5). F0.01(1, 9) values and their probabilities are shown. (DOC) [file pone.0102159.s005.doc]

Table S3. Analysis of variance for pupa aggregation of *D. melanogaster* expressed as index (R ) of aggregation in the presence and in the absence of *D. pavani* larval volatiles (data in Figure 2). Pupa aggregation of *D. pavani*, *D. gaucha* and the hybrids in the presence and in the absence of *D. melanogaster* larval volatiles are also compared (data in Figure 5). F_0.01(1, 9)_ values and their probabilities are shown.

------------------------------------------------------------------------------------------------------------------------

Species and F *P*

strain values

*D- melanogaster*

Oregon R-c 54.23 0.001

Canton – Special 68.75 0.0005

Til – Til 45.67 0.001

Trana 56.49 0.001

*vestigial* 71.83 0.0005

*Or83b*  3.46 0.20

*Syn^97CS^* 4.03 0.10

*rut* 2.76 0.25

*D. pavani*

La Florida 51.78 0.0005

*D. gaucha*

Buenos Aires 2.43 0.25

*D. pavani x D. gaucha* hybrids

*pavani x gaucha* 86.09 0.0005

*gaucha x pavani* 3.12 0.25

F_0.01(1, 9)_ critical value = 13.60, *P* = 0.01
